# Human sperm rheotaxis: a passive physical process

**DOI:** 10.1038/srep23553

**Published:** 2016-03-23

**Authors:** Zhuoran Zhang, Jun Liu, Jim Meriano, Changhai Ru, Shaorong Xie, Jun Luo, Yu Sun

**Affiliations:** 1 Department of Mechanical and Industrial Engineering, University of Toronto, Toronto, ON, Canada; 2 LifeQuest Centre for Reproductive Medicine, Toronto, ON, Canada; 3 Jiangsu Provincial Key Laboratory of Advanced Robotics & Collaborative Innovation Center of Suzhou Nano Science and Technology, Soochow University, China; 4 Department of Mechatronic Engineering, Shanghai University, China; 5 Institute of Biomaterials and Biomedical Engineering, University of Toronto, Toronto, ON, Canada; 6 Department of Electrical and Computer Engineering, University of Toronto, Toronto, ON, Canada

## Abstract

A long-standing question in natural reproduction is how mammalian sperm navigate inside female reproductive tract and finally reach the egg cell, or oocyte. Recently, fluid flow was proposed as a long–range guidance cue for sperm navigation. Coitus induces fluid flow from oviduct to uterus, and sperm align themselves against the flow direction and swim upstream, a phenomenon termed rheotaxis. Whether sperm rheotaxis is a passive process dominated by fluid mechanics, or sperm actively sense and adapt to fluid flow remains controversial. Here we report the first quantitative study of sperm flagellar motion during human sperm rheotaxis and provide direct evidence indicating that sperm rheotaxis is a passive process. Experimental results show that there is no significant difference in flagellar beating amplitude and asymmetry between rheotaxis-turning sperm and those sperm swimming freely in the absence of fluid flow. Additionally, fluorescence image tracking shows no Ca^2+^ influx during sperm rheotaxis turning, further suggesting there is no active signal transduction during human sperm rheotaxis.

In natural fertilization, mammalian sperm travel thousands of their body lengths in the complicated female genital tract to reach the egg cell, or oocyte^1^. The mechanisms of sperm navigation are not well understood; however, it is widely accepted that sperm follow certain guidance cues during the journey^2^,^3^. The known guidance mechanisms are rheotaxis^4^ (i.e., sperm reorient in fluid flow to align against the flow direction and swim upstream), thermotaxis^5^,^6^ (i.e., sperm swim against temperature gradient in oviduct), and chemotaxis^7^,^8^ (i.e., sperm are attracted by chemoattractants secreted by oocyte and its surrounding cumulus cells and swim against the chemical gradient). While chemotaxis and thermotaxis are believed to be active sensing processes^9^,^10^,^11^,^12^, it is still unknown whether rheotaxis involves active flow sensing or not. Although fluid mechanics models have been proposed to explain rheotaxis turning as a passive process^13^, other studies suggest that sperm rheotaxis is an active process where fluid flow is sensed by mechanosentive channels^14^. Both hypotheses lack direct evidence.

In active reorientation such as in chemotaxis and thermtaxis, sperm respond to external stimuli by adjusting their flagellar beating patterns. Both marine invertebrate sperm^15^,^16^ and mammalian sperm^17^,^18^ bend their flagella asymmetrically and swim towards chemoattractants in response to chemical stimulus. Similarly, while turning in temperature gradient, sperm bend their flagella and keep the curved flagella immobile for a short period^19^. These asymmetric flagellar beating patterns are results of microtubule sliding movement regulated by calmodulin and calcium^20^,^21^,^22^,^23^; therefore, active sperm response always comes with calcium signaling and oscillation of intracellular Ca^2+^ concentration ([Ca^2+^]_i_)^24^,^25^,^26^. For rheotaxis reorientation, neither sperm flagellar behavior or [Ca^2+^]_i_ is known.

To understand the mechanism of sperm turning in rheotaxis, this paper presents the first quantitative analysis of human sperm flagellar behavior during rheotaxis turning. The results reveal, both at the single cell and population levels, that there is no significant difference in flagellar beating between rheotaxis turning sperm and free-swimming sperm. Additionally, the constant [Ca^2+^]_i_ signal measured during rheotaxis turning provides further experimental evidence that in contrast to the active process of chemotaxis and thermotaxis, human sperm rheotaxis is a passive process and no flow sensing is involved.

## Methods

### Sperm preparation

Human semen samples were collected, processed and tested in accordance with WHO’s protocol^27^ approved by Research Ethics Board at the University of Toronto, and informed consent was obtained from all subjects. Semen samples were collected from healthy donators and were allowed to liquefy at room temperature (approximately 22 °C) for 30–60 minutes. Motile sperm were purified by the ‘swim-up’ method. Liquefied semen (0.5–1 ml) were layered in a 15-ml falcon tube below 1.5 ml of modified human tubal fluid (mHTF, Irvine Scientific). The falcon tube was loosely tightened and placed in 37 °C water bath for 1 hour to allow for motile sperm to swim up. Supernatant (0.5–1 ml) containing motile sperm was collected and diluted to a density of 2 – 4 × 10^4^ cell/ml for analysis. The low density was specifically chosen to avoid sperm overlap under microscopy imaging. All experiments were completed within 3 hours after sperm collection.

### *In vitro* sperm rheotaxis

**Figure 1:**
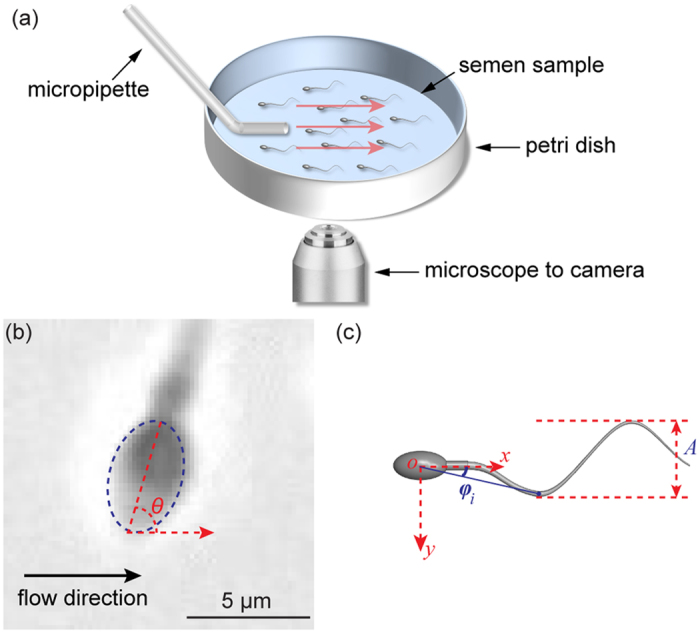
Experimental setup and image analysis of sperm behaviour. (**a**) Schematic of experimental setup (not drawn to scale). Arrows inside the petri dish indicate fluid flow direction. (**b**) Head angle, *θ* is defined as the angle between the major axis (red dashed line) of head ellipse (blue dashed ellipse) and flow direction. (**c**) Flagellar beating is described by the beating amplitude, *A* and asymmetry level, 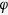. 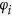 shown in the figure is the angle between the *i*-th point on the flagellum and the horizontal image axis.

Washed sperm (1 ~ 1.5 ml) were placed in a 35-mm petri dish, forming a 1 mm thick liquid film in the petri dish. A micropipette (tip diameter: 125 μm) was placed on the petri dish bottom to generate fluid flow. The micropipette was connected to a 250-microliter syringe mounted on a syringe pump (Pump 11 Pico Plus Elite, Harvard Apparatus) to control the flow velocity. The micropipette and syringe were filled with the same mHTF medium (low viscosity) as used in sperm washing. Both sample and filling mHTF medium were kept at room temperature. Sperm rheotaxis was observed under a standard inverted microscope (TE2000-S, Nikon). A camera (scA 1300–30 gm, Basler Inc.) was connected to the microscope to capture videos under brightfield imaging with a 20× objective (depth of field: 5.8 μm) at 30 frames per second. Only those sperm that remained in focus with a vertical displacement less than 5 μm were chosen for analysis. A schematic diagram of experimental setup is shown in Fig. 1(a).

*In vitro* sperm rheotaxis is affected by fluid flow velocity, shear stress, and fluid viscosity^13^,^14^. Increased fluid flow velocity and shear stress cause more sperm to show rheotactic behaivour, whereas higher fluid viscosity makes sperm rheotaxis turning less efficient. Among these three factors, fluid flow velocity dominates shear stress and viscosity. It was reported that within the flow velocity range of 27 ~ 101 μm/s, the difference in swimming speed between rheotaxis sperm and non-rheotaxis sperm increases with flow velocity^14^, which is consistent with our experimental observation. In our experiments, flow velocity was set constant at approximately 90 μm/s. Around this chosen velocity, flow sensation and intracellular signaling, if existing as proposed in the literature^14^, are most significant. Flow velocity was calibrated to be 92.4 μm/s at the micropipette tip opening. Flow was confirmed to be laminar.

### Measurement of intracellular Ca^2+^

Fluorescent indicator Fluo-4 was used for calcium imaging. Sperm were loaded with 10 μM Fluo-4 AM (Molecular Probes) in the presence of Pluronic F-127 (0.5% v/v, Molecular Probes) at 37 °C for 30–45 minutes. The cells were then centrifuged at 300 g for 5 minutes to remove excess dye. The pellet was re-suspended in 1 ml mHTF medium for analysis. Fluorescence was excited at 488 nm and recorded at 520 nm. Fluorescence imaging was focused on the plane of sperm swimming (approximately 10 μm above the bottom of the petri dish). Fluorescent videos (resolution: 512 × 384 pixels) were captured by a CCD camera (Retiga Exi, Qimaging) under 20X at 20 frames per second. [Ca^2+^]_i_ was expressed as F/F_0_ (%), where F was the intensity of fluorescent signals, and F_0_ was the base intensity which was calculated from averaging the fluorescence intensity of the same sperm swimming freely for one second. Both F and F_0_ were measured from whole sperm cell, including head, midpiece and flagellum.

### Analysis of sperm behavior

Computer vision algorithms were developed to track sperm head and flagellum. Sperm head was segmented directly by thresholding, followed by image thinning to extract the skeleton of the head and midpiece. Sperm flagella were tracked by the maximum intensity region (MIR) algorithm integrated with a Kalman predictor^28^,^29^,^30^, and were represented by discrete points on the flagella. To normalize flagellar beating of sperm swimming towards different directions, the whole sperm was rotated so that the head and midpiece were horizontally facing left [Fig. 1(c)].

Head angle, flagellar beating amplitude, and beating asymmetry were defined to quantitatively describe sperm behavior. Head angle was calculated for monitoring the direction of sperm movement. As shown in Fig. 1(b), head angle *θ* was defined as the angle between the major axis of head ellipse and the horizontal axis of the image. Since the flow direction in experiments was at a 0° angle with the horizontal image axis, the head angle was also referred to as the angle between the sperm head axis and the flow direction. To describe sperm flagella, a Cartesian coordinate was first established with the *x* and *y* axes consistent with image coordinate and origin locating at head centroid [Fig. 1(c)]. Let 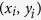 represent the *i*-th point on the flagellum, then the beating amplitude 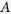 is


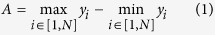


where 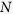 is the total number of points on the flagellum.

Beating asymmetry level 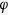 is defined as the average of angle 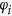, which is the angle between the *i*-th point on the flagellum and the horizontal image axis^31^, and is given by


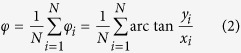


The final results of asymmetry level are converted to degrees.

## Results

### *In Vitro* sperm rheotaxis

**Figure 2:**
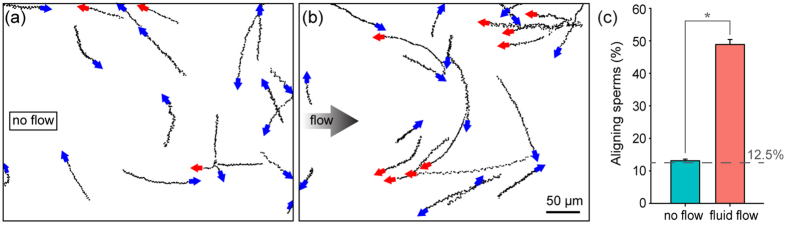
(**a**) Trajectories of free-swimming sperm under the no–flow condition. Rheotaxis sperm are those with head angle within ±22.5° of flow direction (i.e., horizontal image axis), as labeled by red arrows. Blue arrows label non-rheotaxis sperm. (**b**) Trajectories of sperm under fluid flow. (**c**) Percentage of rheotaxis sperm from six independent experiments (n = 342 for no flow and n = 440 for fluid flow condition). *Represents significant difference (p = 7 × 10^−7^ by two tailed t-test).

Figure 2 shows typical sperm trajectories with and without fluid flow. Without fluid flow, sperm swam in random directions with largely linear trajectories [Fig. 2(a)]. However, sperm swam against the artificially generated fluid flow, and curved trajectories were observed, indicating that sperm adjusted their moving directions and gradually aligned against flow [Fig. 2(b)].

To quantify sperm rheotaxis, we summarized the percentage of sperm showing rheotactic movement (hereinafter referred to as rheotaxis sperm). Rheotactic movement was defined as sperm head angle within ±22.5° of flow direction or the horizontal image axis [see sperm trajectories labelled by red arrows in Fig. 2(a,b)]. Since the moving direction of free-swimming sperm is completely random, the theoretical value of the alignment percentage is 12.5% under the no-flow condition (i.e., ±22.5° over ±180°). Our measurement of 342 sperm in six independent experiments demonstrated significant agreement with this theoretical value [13.12 ± 0.46% vs. 12.5%, mean ± sem, Fig. 2(c)]. Under fluid flow, 48.89 ± 1.53% (mean ± sem) of 440 sperm showed rheotactic movement, which was significantly higher than the no-flow condition (p-value: 7 × 10^−7^). These results were consistent with data previously reported in the literature^4^,^14^, confirming that the reorientation behavior observed in our experiments was caused by fluid flow instead of other factors (e.g., chemotaxis^32^ or thermotaxis^5^).

### Unchanging flagellar beating in rheotaxis reorientation

**Figure 3:**
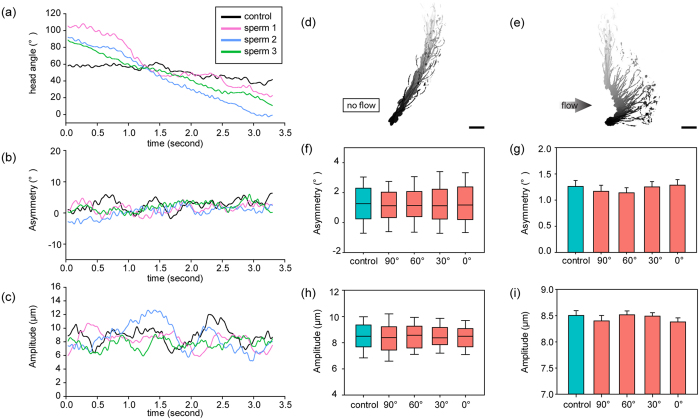
(**a–c**) Quantitative tracking results of head angle, flagellar beating asymmetry, and flagellar beating amplitude of single sperm. A freely swimming control sperm and three representative sperm during rheotaxis reorientation are shown. Fluid flow was turned on at time 0 and kept constant afterwards. (**d**,**e**) Motion history images confirm no active asymmetry flagellum bending for both control sperm under the no-flow condition, and rheotaxis sperm. Scale bar: 10 μm. (**f–i**) Data from a sperm population. n = 15–26 sperm for each data point. Sperm turning process is described by four phases: head angle at 90°, 60°, 30° and 0°. Box plots and mean values of flagellar beating asymmetry (**f**,**g**) and beating amplitude (**h**,**i**) are shown.

Before investigating sperm flagellar beating behaviors during rheotaxis reorientation, we first validated the effectiveness of our measurement method in detecting subtle sperm flagellar beating changes by comparing flagellar beating behaviors of control sperm and hyperactivated sperm (i.e., bourgeonal stimulated sperm^17^, see Supplementary Information for details). We then investigated the flagellar beating behaviors of control and rheotaxis turning sperm. Freely swimming sperm in the absence of fluid flow were selected as the control group. Figure 3(a–c) show quantitative tracking results of the head angle, flagellar beating asymmetry, and beating amplitude of the control sperm and three representative rheotaxis sperm. Time 0 was the moment when fluid flow was turned on and kept constant. Head angle of the control sperm remained constant after fluid flow was turned on, which is consistent with the linear trajectories shown in Fig. 2(a). In contrast, head angles of the rheotaxis sperm decreased significantly, corresponding to the curved trajectories in Fig. 2(b). These two significantly different trends indicate that tracking the head angle can effectively distinguish rheotaxis sperm and monitor rheotaxis status.

Figure 3(b) shows that flagellar beating asymmetry levels of three rheotaxis sperm overlap with that of the control sperm, and all four asymmetry curves fluctuate within ±5°. The fluctuation resulted from sperm wiggling around an averaged path. A sperm swimming freely without any external stimulus does not show active sensation, and thus, does not bend its flagellum asymmetrically for turning, which can be seen in the control sperm data. Overlap in flagellar beating amplitude was also observed [Fig. 3(c)]. Although beating amplitude varied within a beating cycle, both the rheotaxis sperm and control sperm showed the same periodical trend and had a beating amplitude in the same range of 6 μm to 12 μm. These results led us to speculate that there might not be obvious difference in sperm flagellar behavior in the rheotaxis reorientation process.

Analyzing motion history images supported this speculation. As shown in Fig. 3(d,e), sperm silhouettes were captured and overlaid in the same image^33^. Old silhouettes fade with time and thus showing a light gray color while new silhouettes are in dark black color. Fading time was set to 3 sec which was sufficient for recording the complete reorientation process. Without fluid flow, the sperm swam along a straight trajectory in the direction of its head axis with flagella beating periodically around the linear trajectory, as shown in Fig. 3(d). Under fluid flow, the sperm swam in the direction perpendicular to the flow direction at first, and gradually aligned against the flow direction [Fig. 3(e)]. During reorientation, the flagella beat periodically around the head axis in the same way as under the no-flow condition, and no obvious asymmetrically flagellar bending was observed (Supplementary Video 1). These results suggest that sperm flagellar beating asymmetry and amplitude do not change with or without fluid flow.

We then analyzed flagellar beating data collected during rheotaxis reorientation in a sperm population. Sperm head angle was used to monitor reorientation status. Head angles at 90°, 60°, 30°, and 0° were selected to represent the reorientation process from perpendicular to the flow direction (i.e., 90°) to completely aligning against flow (i.e., 0°). At each head angle, flagellar beating asymmetry and amplitude of 15–26 rheotaxis sperm were quantified. Freely swimming sperm were used as the control group. As shown in Fig. 3(f), for each set of data, the three lines of the box show 75 percentile, median, and 25 percentile, and the two whiskers are the maximum and minimum. Medians of flagellar beating asymmetry at each head angle are close to each other, and all values are close to the control group. The 75 and 25 percentile and maximum or minimum values are also close to each other, indicating that all the groups have similar statistical distributions. Mean values of flagellar beating asymmetry are summarized in bar plots in Fig. 3(g). All five groups show a mean asymmetry between 1.0° to 1.5°. Statistical analysis shows no significant difference among groups (p = 0.82 by ANOVA). Analysis of flagellar beating amplitude gives similar results. Freely swimming sperm and rheotaxis sperm at different reorientation status show similar beating amplitude distributions [Fig. 3(h)]. Mean amplitudes are all around 8.5 μm [Fig. 3(i)] and no significant difference is found (p = 0.66 by ANOVA). These results from a sperm population, together with results of single sperm, indicate that sperm do not change their flagellar beating behavior (i.e., amplitude and asymmetry level) during rheotaxis reorientation, suggesting sperm rheotaxis might not involve active sensation.

### No active signaling transduction in rheotaxis reorientation

Calcium plays a central role in sperm signaling^25^. Calcium binds to the calmodulin in the 9 + 2 axoneme structure of the dynein motor which generates sperm motion, thus linking intracellular signaling to changes in flagellar bending and sperm motion^34^,^35^. Almost all sperm activities (e.g., capacitation^36^, acrosome reaction^37^, hyperactivation^38^, and chemosensation^39^) include calcium in their signaling pathway. Although other molecules^40^ and ions^41^ are also involved in active intracellular signaling, activities of these molecules or ions are always followed by the oscillation of [Ca^2+^]_i_^42^,^43^. Therefore, we monitored [Ca^2+^]_i_ of rheotaxis sperm to further investigate whether there is active signal transduction in sperm rheotaxis.

**Figure 4:**
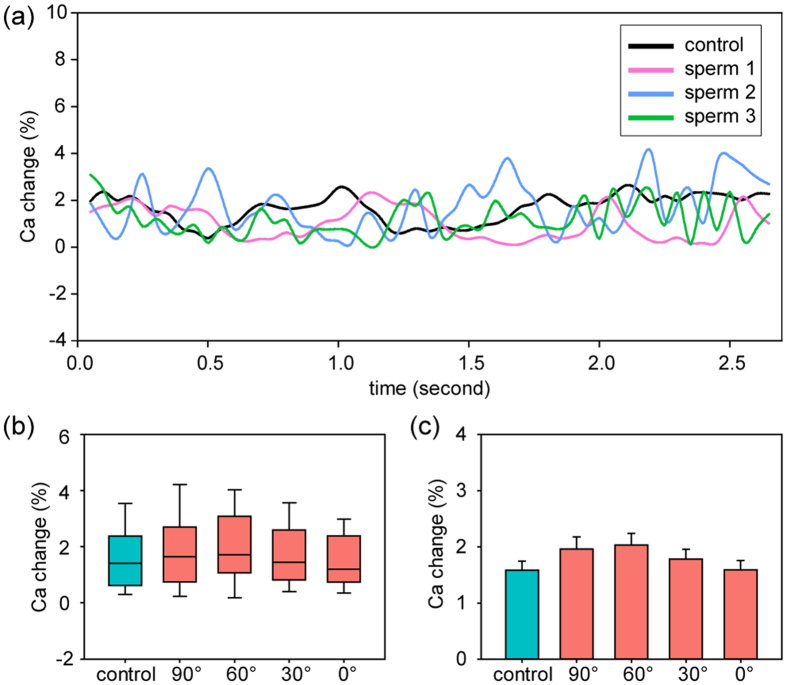
(**a**) Measurement of [Ca^2+^]_i_ of single sperms during sperm rheotaxis reorientation. Fluid flow was turned on at 0 sec and kept constant afterwards. (**b**) Box plot and (**c**) mean values show that no [Ca^2+^]_i_ changes exist throughout the sperm turning process. n = 10 sperm for each data.

Measuring dynamic [Ca^2+^]_i_ of motile sperm is challenging when sperm move in and out of the focal plane, which can greatly change fluorescence intensity. As shown in Fig. 4(a), [Ca^2+^]_i_ of a control sperm fluctuates within 4%. Compared to the more than 10% of [Ca^2+^]_i_ increase in active sensation reported in the literature^10^,^44^,^45^, our measurement results indicate that the influence of sperm movement including deviations from the focal plane is negligible.

Figure 4(a) shows three representative rheotaxis sperm revealing the same [Ca^2+^]_i_ as control sperm. Although fluctuating, all [Ca^2+^]_i_ data curves present a change of less than 5%, suggesting no significant change in [Ca^2+^]_i_ during rheotaxis reorientation. In Fig. 4(b), the medians of control sperm and rheotaxis sperm at different head angles are less than 2%, and the maximum [Ca^2+^]_i_ changes are less than 4%. The minimums of all five groups are close to zero, confirming that, rather than induced by intracellular signal transduction, fluctuation in data mainly resulted from sperm’s wiggly movement. Mean values of [Ca^2+^]_i_ change are around 2%, and no significant difference was found among the groups [p = 0.3 by ANOVA, see Fig. 4(c)]. These results suggest that no calcium signaling occurs in the process of rheotaxis reorientation, and explain the measurement results presented in the section of ‘Unchanging flagellar beating in rheotaxis reorientation’. The measured constant [Ca^2+^]_i_ data also indicate that sperm rheotaxis does not involve other active sperm activities such as capacitation and hyperactivation.

## Discussion

To investigate whether sperm rheotaxis is an active or passive process, this work quantitatively reveals that no significant difference exists in both flagellar beating (beating amplitude and asymmetry) and [Ca^2+^]_i_ between rheotaxis sperm and control sperm. The possibility that extremely weak intracellular signaling might exist and sperm might adjust its flagellar beating with extreme subtleties during rheotaxis reorientation, cannot be excluded. However, the difference is insignificant between rheotaxis sperm and freely swimming sperm. Therefore, our results indicate that human sperm rheotaxis is a passive physical process resulting from hydrodynamic interactions between sperm flagellum and its surrounding fluid flow.

**Figure 5:**
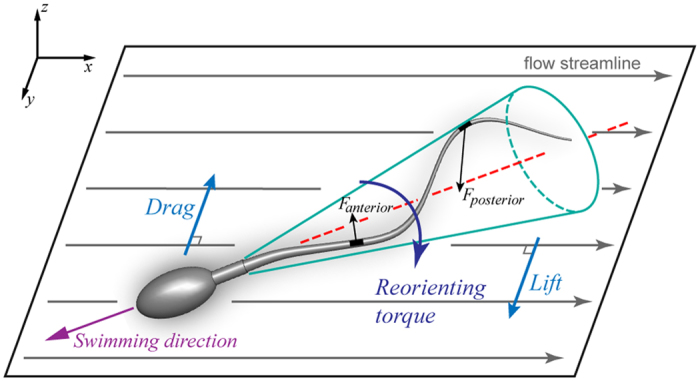
Schematic showing passive sperm reorientation. Sperm flagellum is approximated by a chiral helix with a conical envelope. In fluid flow, sperm swims with an angle to the flow streamline. The chirality and imbalance of hydrodynamic force on posterior and anterior flagellum produce a net lift force, which is opposed by the drag force on sperm, resulting in a torque reorienting sperm upstream.

As illustrated in Fig. 5, sperm flagellar beating can be approximated by a conical helix^13^. Encountering fluid flow, the sperm keeps its original flagellar beating. However, due to the conical shape and the chirality of sperm flagellum, posterior flagellum receives stronger hydrodynamic force than the anterior, resulting in a net lift force perpendicular to the flow direction^4^,^46^. The lift force is balanced by the drag force, generating a torque which reorients sperm upstream. Sperm is subjected to the reorienting torque and therefore, presents rheotactic behaviour. The helical beating shape of sperm flagella plays a central role in this mechanism, and is also the basis for determining the direction of sperm rheotactic turning after the reversal of flow direction^47^.

It has been hypothesized that calcium entry via CatSper channels is essential for sperm rheotaxis^4^. CatSper channels are sperm-specific Ca^2+^ channels located on sperm flagellum^48^,^49^. In response to chemical stimulus, CapSper channels mediate Ca^2+^ influx directly from extracellular medium into flagellum and simultaneously change flagellar beating^50^,^51^. However, no calcium influx at flagella was observed in this work, and [Ca^2+^]_i_ of whole sperm remained constant in sperm rheotaxis (Supplementary Video 2), suggesting that CatSper channels play potential roles in rheotaxis by a different mechanism rather than chemosensation, which requires further investigation.

As a passive guidance mechanism, rheotaxis might govern sperm behavior in a more general manner than previously thought. It was suggested that besides three-dimensional flagellar beating, planar flagellar beating might also reveal rheotactic behavior^52^. Recently, the planar slither swimming mode of sperm near surface was discovered^53^, suggesting rheotaxis may not only lead sperm’s direction in central oviduct, but also guide sperm near oviduct wall. Moreover, rheotaxis may also guide sperm inside the uterus. Since coitus induces fluid secretion in the oviduct^4^, it is rational to speculate that these fluids generate fluid flow along the entire reproductive tract to provide guiding cues for sperm.

There could be many reasons why nature designs rheotaxis as a passive process. For instance, as long-range guidance, rheotaxis requires a highly robust guiding mechanism. In terms of guiding cues, generating fluid flow is easier and more stable than generating a gradient (e.g., chemical or temperature gradient) where each single point along the path needs to be accurately controlled. With respect to guiding responses, a passive physical process induces higher percentage of responsive sperm than active sensation (approximately 50% sperm showing rheotaxis^4^,^14^ compared to less than 10% showing chemotaxis^54^ and thermotaxis^5^). Flagellar beating has much less strict requirements than an intact intracellular signal pathway where multiple molecules are involved, and every biochemical reaction step must be fully functional^2^,^55^. Regarding energy dissipation, a passive physical process does not cost biochemical energy that is required in active sensations such as chemotaxis and thermotaxis. A sperm has unique metabolism and needs to schedule/allocate its energy consumption cautiously^56^,^57^. Without extra energy consumption in rheotaxis, sperm can reserve energy for traveling the long journey to the oocyte and for executing other important tasks such as acrosome reaction to penetrate the zona pellucida for eventual fertilization.

## Additional Information

**How to cite this article**: Zhang, Z. *et al*. Human sperm rheotaxis: a passive physical process. *Sci. Rep*. **6**, 23553; doi: 10.1038/srep23553 (2016).

## Supplementary Material

Supplementary Information

Supplementary Video S1

Supplementary Video S2

Supplementary Video S3

Supplementary Video S4
