# Supplementary material for: Human sperm rheotaxis: a passive physical process
Source: Sci Rep. 2016 Mar 23;6:23553. doi: 10.1038/srep23553 (PMC4804285; doi:10.1038/srep23553)
Supplement: Supplementary Information [file srep23553-s1.pdf]

Human sperm rheotaxis: a passive physical process

Zhuoran Zhang[1], *Jun Liu*[1], *Jim Meriano*[2], *Changhai Ru*[3], *Shaorong Xie*[1,4], *Jun Luo*[1,4], *and Yu Sun*[1,5,6*]

[1]Department of Mechanical and Industrial Engineering, University of Toronto, Toronto, ON, Canada

[2]LifeQuest Centre for Reproductive Medicine, Toronto, ON, Canada

[3]Jiangsu Provincial Key Laboratory of Advanced Robotics & Collaborative Innovation Center of Suzhou Nano Science and Technology, Soochow University, China

[4]Department of Mechatronic Engineering, Shanghai University, China

[5]Institute of Biomaterials and Biomedical Engineering, University of Toronto, Toronto, ON, Canada

[6]Department of Electrical and Computer Engineering, University of Toronto, Toronto, ON, Canada

*Corresponding author:

Yu Sun

E-mail: sun@mie.utoronto.ca

Tel: 1-416-946-0549

Fax: 1-416-978-7753

Supplementary Information

To validate the effectiveness of our method in detecting the subtle sperm flagellar beating changes, we performed the same analysis on hyperactivated sperm because hyperactivation is known to cause sperm flagellar beating changes. In the experiments, sperm were stimulated by bourgeonal, a molecule capable of inducing asymmetric flagella beating and Ca^{2+} influx[1], and stimulated sperm presented hyperactivated motility (Supplementary Video 3). Quantitative tracking results confirmed significantly different flagellar behavior. Flagellar beating asymmetry of bourgeonal stimulated sperm oscillated more than $\pm 30°$ while that of control sperm and rheotaxis turning sperm fluctuated only within $\pm 5°$ ($p<0.0001$, Supplementary Figure S1). Bourgeonal stimulated sperm also showed significantly higher flagellar beating amplitude ($p<0.0001$, Supplementary Figure S1). These are in agreement with characteristics of hyperactivated motility including highly asymmetric flagellar beating[2] and larger beating amplitude[3], indicating that our analysis technique is capable of distinguishing different flagellar beating patterns.

As for calcium measurement, the calcium-sensitive dye (Fluo-4) used in this work has been proven that it is capable of monitoring dynamic $[Ca^{2+}]_i$ oscillation since the binding and unbinding of Ca^{2+} to fluorescent indicator occurs in milliseconds while calcium signaling occurs on the order of seconds[4]. Therefore, the dynamic Ca^{2+} signals are not compensated by the kinetics of the dye[5]. Additionally, in bourgeonal stimulation experiments, stimulated sperm demonstrate obvious Ca^{2+} influx and significantly higher $[Ca^{2+}]_i$ than control sperm, which is in agreement with the literature[6]. Fluorescence intensity increase at sperm flagellum was also observed in bourgeonal-stimulated sperm (Supplementary Video 4). These results confirm the capability of our experimental setup for detecting dynamic calcium signaling.

Bourgeonal stimulation methods

Bourgeonal (Enzo Life Sciences, Inc.) was injected into HEPES buffered modified human tubal fluid medium (mHTF, Irvine Scientific) to stimulate sperm. Before injection, an injection micropipette pulled from a glass capillary was modified to form a 1 μm tip. Injection micropipette was filled with 20 μL

bourgeonal (1mM in DMSO), and connected to a digitally controlled pump (XenoWorks Digital Microinjector, Sutter Instrument). During injection, the injection micropipette was brought into central field of view in the same focal plane of motile sperm. Injection pressure was chosen such that the injecting volume was comparable with sperm volume and the injected fluid did not form turbulence which disturbed sperm motion. Injected bourgeonal generated the gradient by passive diffusion. Sperm swimming towards the injection position were chosen for analysis. Images were captured in brightfield under 20X objective at 30 frames per second.

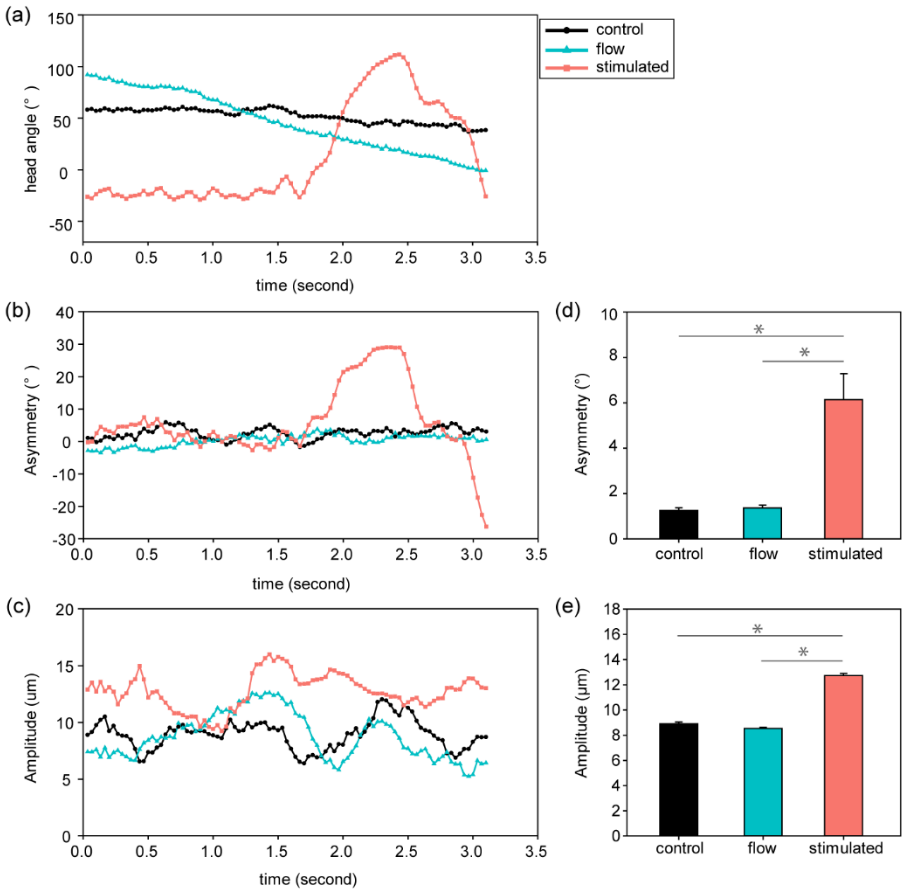

Supplementary Figure S1. Quantitative results of sperm head angle (a), flagellar beating asymmetry level (b), and flagellar beating amplitude (c) of a freely swimming sperm as control, a rheotaxis sperm, and a bourgeonal stimulated sperm. Stimulated sperm shows hyperactivated motility (Supplementary Video 3). Stimulated sperm show significantly higher flagellar beating asymmetry levels (d) and beating amplitude (e) than control and rheotaxis sperm, confirming the effectiveness of our algorithms in detecting subtle changes in flagellar beating. For (d) and (e), each data point represents mean value of 10 sperm. Error bar is standard error of mean. * represents significant difference ($p<0.001$ by two-tailed t-test).

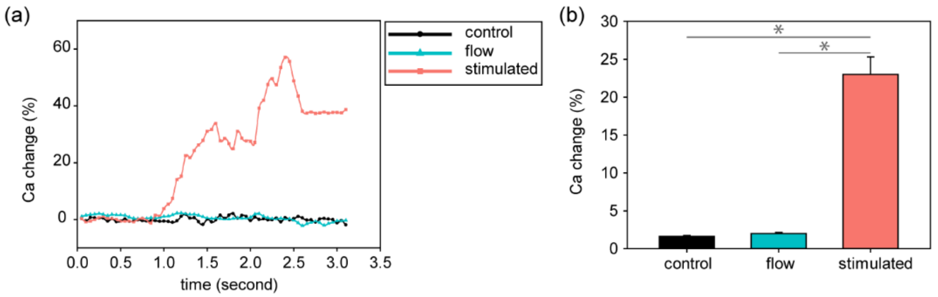

Supplementary Figure S2. (a) Fluorescent tracking results of $[Ca^{2+}]_i$ of a control sperm, rheotaxis sperm and bourgeonal stimulated sperm. (b) Stimulated sperm show significant Ca^{2+} influx (Supplementary Video 4) and higher $[Ca^{2+}]_i$ than control and rheotaxis sperm, confirming our experimental setup is capable of detecting changes in $[Ca^{2+}]_i$. Each data point represents mean value of 10 sperm. Error bar is standard error of mean. * represents significant difference ($p<0.001$ by two-tailed t-test).

Supplementary Video 1. Sperm showing rheotaxis turning.

Supplementary Video 2. Ca imaging of sperm showing rheotaxis turning.

Supplementary Video 3. Stimulated sperm showing hyperactivated motility.

Supplementary Video 4. Ca imaging of stimulated sperm showing hyperactivated motility.

Supplementary References

1 Spehr, M. *et al.* Identification of a Testicular Odorant Receptor Mediating Human Sperm Chemotaxis. *Science* **299**, 2054-2058 (2003).

2 Quill, T. A. *et al.* Hyperactivated sperm motility driven by CatSper2 is required for fertilization. *Proceedings of the National Academy of Sciences* **100**, 14869-14874 (2003).

3 Suarez, S. S. Hyperactivated Motility in Sperm. *Journal of Andrology* **17**, 331-335 (1996).

4 Faas, G. C., Raghavachari, S., Lisman, J. E. & Mody, I. Calmodulin as a direct detector of Ca2+ signals. *Nat Neurosci* **14**, 301-304 (2011).

5 Alvarez, L. *et al.* The rate of change in Ca(2+) concentration controls sperm chemotaxis. *The Journal of cell biology* **196**, 653-663 (2012).

6 Spehr, M. *et al.* Particulate Adenylate Cyclase Plays a Key Role in Human Sperm Olfactory Receptor-mediated Chemotaxis. *Journal of Biological Chemistry* **279**, 40194-40203 (2004).
